# Social Risk at Individual vs Neighborhood Levels and Health Care Use in Medicaid Enrollees

**DOI:** 10.1001/jamanetworkopen.2025.5047

**Published:** 2025-04-15

**Authors:** Margae J. Knox, Emma L. Tucher, Chris Miller-Rosales, Jodi McCloskey, Richard W. Grant, Esti Iturralde

**Affiliations:** 1 Division of Research, Kaiser Permanente Northern California, Pleasanton, California; 2 Analysis Group, Menlo Park, California

## Abstract

**Importance:**

Health-related social risks are increasingly recognized as important contributors to health. Compared with individual screening, neighborhood measures are potentially a lower cost, scalable strategy for identifying social risk.

**Objective:**

To inform health resource planning and social risk screening strategies by comparing self-reported vs neighborhood-level social risk with inpatient, emergency department (ED), and outpatient care.

**Design, Setting, and Participants:**

A cross-sectional analysis of self-reported social risks measured during Medicaid enrollment and neighborhood-level social risk in relation to health care use was conducted. Members of Kaiser Permanente Northern California, a large integrated health care delivery system, who completed the Medicaid Integrated Outcomes Questionnaire from January 1, 2018, to February 29, 2020, were included. Analysis took place from January 8 to November 29, 2024.

**Exposure:**

Neighborhood-level social risk (living in the least-resourced Neighborhood Deprivation Index quartile) and self-reported social risk (indicating a need or wanting help with finances, food, housing, or transportation domains).

**Main Outcomes and Measures:**

Hospital and ED admissions, primary care, specialty care, mental health, and social work visits in the year prior to questionnaire completion. Multivariable negative binomial regression models were analyzed for each type of health care use, controlling for demographic characteristics and several health conditions (eg, asthma, hypertension, and chronic pain).

**Results:**

Among 13 527 respondents (8631 [63.8%] female; 5289 [39.1%] aged 25-44 years; 2846 [21.0%] Asian, 1986 [14.7%] Black or African American, 3040 [22.5%] Hispanic, 4602 [34.0%] White, and 1053 [7.8%] other race or ethnicity), 33.8% in the most-resourced neighborhood reported at least 1 social risk vs 40.1% in the least-resourced quartile (*P* < .001). Individual- and neighborhood-level measures were each associated with ED visits (marginal effect estimate for both measures: 0.23; 95% CI, 0.17-0.29). Neither measure was associated with hospital admissions. Individual risk was associated with greater use of all outpatient services (ranging from primary care visit marginal effect estimate: 0.22; 95% CI, 0.13-0.31 to mental health visit marginal effect estimate: 1.21; 95% CI, 0.67-1.75). Neighborhood-level risk was not associated with most outpatient visits and was negatively associated with mental health visits.

**Conclusions and Relevance:**

In this cross-sectional study, associations were found for hospital and ED use but not outpatient visits, especially mental health visits. These findings suggest that individual social risk screening appears to provide distinct information compared with neighborhood social risk.

## Key Points

**Question:**

How do neighborhood-based social risk measures compare with self-reported social risk measures in identifying inpatient, emergency department, and outpatient care use in Medicaid enrollees?

**Findings:**

In this cross-sectional study of individual- vs neighborhood-based social risk including 13 527 respondents, both measures of risk were associated with more emergency department visits but were not associated with hospital visits. Individual-level measurement was associated with more primary care, specialty care, mental health, and social work visits, and neighborhood-based measurement was associated with fewer mental health visits but not with other outpatient visits.

**Meaning:**

The findings of this study suggest that social risk screening during Medicaid enrollment yields valuable information that is distinct from neighborhood-level measurement.

## Teaser

This cross-sectional study compares the use of self-reported vs neighborhood-level screening measures to aid in strategies addressing social risk.

## Introduction

The social determinants of health—or factors outside the health care setting, such as economic opportunities, neighborhood resources, and social connections—have well-established associations with morbidity, mortality, and other health indicators.^1,2,3,4,5,6^ Thus, to better support patient health, an increasing number of new care guidelines and performance measures^7,8^ recommend that health systems screen patients for social risks. Social risks are specific adverse social conditions including financial strain, food insecurity, housing barriers, or transportation barriers.^9^ In one large integrated delivery system, most patients reported experiencing one or more social risks.^10^ Research on health care use modeling has found that including social risk measurements can improve model performance,^11,12^ suggesting that social risk information may be valuable data to proactively identify patients for care coordination, outreach, and other supplemental support.

Despite emerging regulatory pressure^7,8^ and strong evidence linking social risks with worse health care outcomes,^13,14^ systematic social risk screening has not been widely adopted.^15,16^ Many clinicians and frontline staff find the screenings resource intensive and difficult to implement.^17^ Furthermore, patient acceptability of social risk screening varies. Less than half of patients surveyed in a large integrated delivery system agreed that health systems should ask about social needs.^18^ In addition, although screening was generally acceptable among patients in settings focused on public and uninsured populations, many expressed discomfort with including social risk information in electronic health records.^19^ Important implementation concerns include stigma from disclosing needs, patient disappointment when resources to address needs are unavailable, and other unintended consequences.^20,21,22^

Neighborhood-based measurement as a proxy for individual social risks represents a low-cost scalable alternative to direct screening that does not require new workflows or overcoming patient hesitancy to share information. As with individual social risks, neighborhood-based indices, such as the Neighborhood Deprivation Index (NDI),^23^ have been associated with various health outcomes, including blood pressure control, preterm birth rates, mental health, and mortality.^24,25,26,27^ However, prior studies have found only modest differences between individual social risks and neighborhood-level socioeconomic status, and misclassification is common when directly inferring individual need from neighborhood measures.^28,29,30^ In one study, for example, 2 of 5 patients experiencing at least 1 social risk did not live in a low-resourced neighborhood.^31^

Further work is needed to better understand how neighborhood-based and individual social risks from the same population may relate to other outcomes, despite measurement differences. Prior research in Medicare populations has compared individual- and neighborhood-level social risk factors with expenditure outcomes.^32,33,34^ To our knowledge, no large studies to date have examined health care use in Medicaid populations, where new comprehensive social risk support interventions are proliferating, especially under Medicaid 1115 waivers.^35^ To our knowledge, associations across a range of health services, including outpatient primary care, mental health, and social work support, have not been documented.

Understanding how individual- and neighborhood-level social risk measurement for the same population relate to various health care use measures can inform trade-offs of using each data type in health services modeling. Insights may help identify whether collecting and analyzing individual-level social risk data are likely to yield unique insights compared with more easily integrated neighborhood measures. Insights may also help improve the design of care coordination programs to support patients with complex health and social needs, shedding light on individual- vs neighborhood-level data utility in population identification, outreach, and intervention strategies.

This study compares self-reported social risks by neighborhood deprivation quartile among a population of Medicaid enrollees who completed a comprehensive social risk questionnaire. It then compares self-reported social risks and neighborhood deprivation quartile with key health services: hospitalizations, emergency department (ED) visits, primary care visits, specialty visits, mental health visits, and social work visits. We hypothesized that individual- and neighborhood-level measures will have directionally similar positive associations with health care use, with higher incident rate ratios between individual social risk and health care use.

## Methods

### Study Design and Setting

This study was a cross-sectional analysis of self-reported social risks, neighborhood deprivation, and health care use conducted within Kaiser Permanente Northern California (KPNC). Kaiser Permanente Northern California is an integrated health care delivery system that provides care to over 4.5 million members insured through employer-based plans, Medicare Advantage, Medicaid, and the California health insurance exchange. Demographic characteristics of KPNC members are similar to the general population in Northern California.^36^ The study was reviewed and approved by the KPNC Institutional Review Board with a waiver of informed consent because of the minimal risk of harm to participants. We followed the Strengthening the Reporting of Observational Studies in Epidemiology (STROBE) reporting guideline.^37^

### Participants

Participants included 13 649 KPNC members who completed the Medi-Cal Integrated Outcomes Questionnaire between January 1, 2018, and February 29, 2020. The questionnaire was administered as part of standard Medicaid enrollment processes and included items assessing both the presence of social risks and individuals’ interest in receiving help across multiple domains (Table 1). Phone outreach workers made at least 2 phone call attempts to each enrollee to collect responses, per state requirements. Administrative leaders estimate that about 50% of enrollees completed the questionnaire. However, because the questionnaire was for operational and not research purposes, response rates were not systematically tracked. While characteristics of all members offered the questionnaire cannot be assessed, basic demographic traits of Medicaid members with at least 1 outpatient visit during a similar timeframe are provided as a proxy comparison (eTable 4 in Supplement 1).

**Table 1.** Screening Questions

Risk domain	Initial outcomes questionnaire		Positive for risk (yes to any question in the domain)
	Question^a^	Checkbox response option	
Financial strain	I’m going to read you a short list of things that people sometimes have trouble paying for. Please tell me whether in the past 3 mo, you have had trouble paying for any of these things. (select all that apply)	Healthy food (ie, fruits and vegetables) Housing Heat and electricity Medical needs Transportation Childcare Debts None of these Other Skip	One or more checked (excluding skip, none of these, and prefer not to answer)
Food insecurity	In the past 3 mo, how often have you worried that your food would run out before you had money to buy more?	Never Sometimes Often Very often Skip	Sometimes, often, or very often
Food insecurity	I’m going to read you a list of things that people sometimes need help with. You may not need or want help with any of these things. However, if you do, we may be able to suggest ways to get the help you need. Which of the following would you like to receive help with at this time?^b^ (Select all that apply)	Food Housing Transportation Utilities Medical Care, medicine, medical supplies Dental services Vision services Applying for public benefits More help with activities of daily living Childcare, other child-related issues Debt, loan repayment Legal issues Employment Other I don’t want help with any of these Skip	Food
Housing barriers/Insecurity	Do you have any concerns about your current living situation, such as housing conditions, ability to pay for housing or utilities, feeling safe, lack of more permanent housing, or something else?	Yes No	Yes
Housing barriers/insecurity	What is your current living situation? (Select 1 only)	Live alone in my own home (house, apartment, condo, trailer, etc); may have a pet Live in a household with other people Live in a residential facility where meals and household help are routinely provided by paid staff (or could be if requested) Live in a facility such as a nursing home which provides meals and 24-hour nursing care Temporarily staying with a relative or friend Temporarily staying in a shelter or homeless Other, describe	Temporarily staying with friend/relative or Temporarily staying in shelter or homeless
Lack of transportation	Has lack of transportation kept you from medical appointments or from doing things needed for daily living? (Select all that apply)	Kept me from medical appointments or from getting medications Kept me from doing things needed for daily living Not a problem for me	Kept me from medical appointments or from getting medications or Kept me from doing things needed for daily living
Lack of transportation	I’m going to read you a list of things that people sometimes need help with. You may not need or want help with any of these things. However, if you do, we may be able to suggest ways to get the help you need. Which of the following would you like to receive help with at this time?^b^ (Select all that apply)	Food Housing Transportation Utilities Medical care, medicine, medical supplies Dental services Vision services Applying for public benefits More help with activities of daily living Childcare, other child-related issues Debt, loan repayment Legal issues Employment Other I don’t want help with any of these Skip	Transportation

^a^ Questions are presented verbatim.

^b^ “Which of the following would you like to receive help with at this time?” question was asked only once but is repeated in the table for clarity. While many needs were elicited, this analysis prioritized food, housing, and transportation needs.

All member demographic and clinical characteristics were sourced from KPNC’s Virtual Data Warehouse, which standardizes and aggregates health record data across the health care system.^38^ Data on social work visits and the social risk questionnaire responses were sourced directly from the electronic health record. Members in this analysis were required to have 6 months or more of health plan enrollment before the questionnaire completion to allow adequate measurement of health care use.

### Variables

#### Individual Level

Our primary measure for assessing individual social risk was based on a positive response from the questionnaire to any of 4 priority areas: financial strain, food insecurity, housing barriers, or transportation barriers. Questionnaire items mirror the previously published “Your Current Life Situation” social needs screening.^39,40^ They have high agreement with the established Accountable Health Communities screening tool and similar validity in relation to self-rated health.^41^ While social risk and social need are distinct concepts,^9^ we consistently refer to positive responses as social risks even though our measures could include both risks and needs (ie, questions about the presence of social factors and about interest in support).

#### Neighborhood Level 

We assessed neighborhood-based social risk by assigning a census tract–level NDI score using the enrollee’s address at the time of survey completion. The NDI is a composite of 8 community-level dimensions, including income, educational level, occupation, and housing conditions of the community, drawing from the American Communities Survey 5-year estimates.^23^ We used the index measure rather than specific neighborhood measurements of social determinants of health given the index’s broad adoption and breadth of existing research relating the index to important health outcomes.^23,42,43,44,45^ The NDI was segmented into quartiles, anchored to neighborhood deprivation scores across the full KPNC membership. As the dependent variable, health care use was measured as the number of hospitalizations and visits with ED, primary care, specialty care, behavioral health, and social work services in the 12 months before questionnaire completion.

#### Covariates

Member demographic characteristics included sex (male and female), age (18-24, 25-44, 45-64, ≥65 years), race and ethnicity (Asian, Black or African American, Hispanic, White, and other race or ethnicity), need for interpreter, and current tobacco use. Race and ethnicity are derived from the electronic health record and based on an algorithm that first assigns a race and ethnicity category based on self-report, then uses administrative data collected by health care professionals or staff if no self-reported data are available. Race responses were not used for those reporting Hispanic ethnicity. The other race or ethnicity category includes Native American individuals due to small sample sizes, as well as unknown and other designations. While race and ethnicity are social constructs without biological meaning, we include these traits in our analyses as potential indicators of structural racism experiences, including exclusion from health-promoting opportunities.

Covariates also included several health conditions common among KPNC members with high-cost health care use, including anemia; asthma; chronic kidney disease; diabetes; hyperlipidemia; hypertension; rheumatoid arthritis and osteoarthritis; fibromyalgia, chronic pain, and fatigue; migraine and chronic headache; obesity; peripheral vascular disease; mental health disorders (depression, anxiety, personality disorder, posttraumatic stress disorder, bipolar disorder, and schizophrenia or schizoaffective conditions); and substance use disorders (alcohol, opioid, and other drug use disorders).

### Statistical Analysis

All statistical analyses were conducted with Stata, version 18 (StataCorp LLC) between January 8 and November 29, 2024. We first descriptively analyzed the study sample and characterized differences in member social risks by quartile of the NDI. We then dichotomized neighborhood deprivation by the highest NDI quartile (least resourced) vs all others, consistent with past research.^29^ Using separate negative binomial models for each type of health care use, we examined health care use and social risks, first modeling possible associations with neighborhood-level need alone, then any individual social risk alone, and finally modeling both measures of social risk together. All models controlled for demographic and health condition covariates. For the final model, incident rate ratios (IRRs) were converted to marginal effect estimates, interpreted as the difference in number of visits should a member shift into the highest NDI (least resourced) quartile or from having no social risks to any social risk. We did not apply survey weights or further analytic adjustments. To examine specific social risks, multivariate modeling was repeated for each need (finances, food, housing, or transportation) as an independent variable for each outcome.

## Results

 Of 13 649 members with 6 months of membership data, 95 (0.7%) were missing a response to any social risks question domain (n = 95 for financial risk, n = 67 for food insecurity, n = 56 for housing insecurity, and n = 78 for transportation barriers). Address data to link NDI scores was missing for 27 members (0.2%). We conducted complete case analysis, with a total sample size of 13 527 patients.

Of the 13 527 members included in the sample, 8631 (63.8%) were female and 4896 (36.3%) were male; 13.8% of all participants were aged 18 to 24 years, 39.1% were 25 to 44 years, 31.5% were 45 to 64 years, and 15.6% were 65 years or older (Table 2). In terms of race and ethnicity, 2846 members (21.0%) were Asian, 1986 (14.7%) were Black or African American, 3040 (22.5%) were Hispanic, 4602 (34.0%) were White, and 1053 (7.8%) were of other race or ethnicity. A total of 5746 members (42.5%) in the sample lived in a neighborhood in the least-resourced quartile, compared with the neighborhood distribution for the full KPNC membership. Members in the least-resourced neighborhood quartile compared with those in other neighborhoods were 65.5% vs 62.6% female (*P* < .001), had similar age groupings (eg, 38.3% vs 39.7% aged 25-44 years; *P* = .11), and had a race and ethnicity distribution of 18.1% vs 23.2% Asian, 21.5% vs 9.6% Black or African American, 27.6% vs 18.7% Hispanic, 25.3% vs 40.5% White, and 7.5% vs 8.0% other race or ethnicity (*P* < .001). Members in the least-resourced neighborhood quartile compared with other neighborhoods also had a greater prevalence of health diagnoses, including hypertension (31.8% vs 25.2%; *P* < .001), diabetes (20.0% vs 14.7%; *P* < .001), obesity (24.1% vs 16.7%; *P* < .001), and fibromyalgia, chronic pain, and fatigue (20.2% vs 15.4%; *P* < .001). Mental health and substance use diagnoses among members in the least-resource neighborhoods compared with others were 20.8% vs 19.2% for depression (*P* = .02), 19.4% vs 20.2% for anxiety (*P* = .03), 5.9% vs 5.1% for bipolar disorder (*P* = .04), and 9.2% vs 6.9% for drug or opioid use disorder diagnoses (*P* < .001). Characteristics by self-reported social risk are reported in eTable 1 in Supplement 1.

**Table 2.** Study Population

Characteristic	No. (%)		
	Total (N = 13 527)	More-resourced neighborhoods (lower 3 NDI quartiles) (n = 7781)	Least-resourced neighborhoods (top NDI quartile) (n = 5746)
Demographics			
Sex			
Female	8631 (63.8)	4868 (62.6)	3763 (65.5)
Male	4896 (37.2)	2913 (34.8)	2001 (35.5)
Age, y			
18-24	1870 (13.8)	1087 (14.0)	783 (13.6)
25-44	5289 (39.1)	3087 (39.7)	2202 (38.3)
45-64	4256 (31.5)	2383 (30.6)	1873 (32.6)
≥65	2112 (15.6)	1224 (15.7)	888 (15.5)
Race and ethnicity^a^			
Asian	2846 (21.0)	1807 (23.2)	1039 (18.1)
Black or African American	1986 (14.7)	750 (9.6)	1236 (21.5)
Hispanic	3040 (22.5)	1453 (18.7)	1587 (27.6)
White	4602 (34.0)	3148 (40.5)	1454 (25.3)
Other^b^	1053 (7.8)	623 (8.0)	430 (7.5)
Needs interpreter	1150 (8.5)	563 (7.2)	587 (10.2)
Tobacco use: current	1485 (11.0)	707 (9.1)	778 (13.5)
Diagnoses			
Anemia	1396 (10.3)	700 (9.0)	696 (12.1)
Asthma	2106 (15.6)	1130 (14.5)	976 (17.0)
Chronic kidney disease	1369 (10.1)	662 (8.5)	707 (12.3)
Diabetes	2290 (16.9)	1142 (14.7)	1148 (20.0)
Hyperlipidemia	2950 (21.8)	1583 (20.3)	1367 (23.8)
Hypertension	3786 (28.0)	1960 (25.2)	1826 (31.8)
Acquired hypothyroidism	985 (7.3)	543 (7.0)	442 (7.7)
Rheumatoid arthritis/osteoarthritis	1815 (13.4)	924 (11.9)	891 (15.5)
Fibromyalgia, chronic pain, and fatigue	2363 (17.5)	1202 (15.4)	1161 (20.2)
Migraine and chronic headache	1206 (8.9)	666 (8.6)	540 (9.4)
Obesity	2680 (19.8)	1298 (16.7)	1382 (24.1)
Peripheral vascular disease	1567 (11.6)	798 (10.3)	769 (13.4)
Depression	2691 (19.9)	1494 (19.2)	1197 (20.8)
Anxiety	2686 (19.9)	1570 (20.2)	1116 (19.4)
Personality disorder	363 (2.7)	203 (2.6)	160 (2.8)
Bipolar disorder	730 (5.4)	393 (5.1)	337 (5.9)
Posttraumatic stress disorder	381 (2.8)	207 (2.7)	174 (3.0)
Schizophrenia or schizoaffective conditions	431 (3.2)	232 (3.0)	199 (3.5)
Alcohol use disorder	292 (2.2)	169 (2.2)	123 (2.1)
Drug or opioid use disorder	1066 (7.9)	536 (6.9)	530 (9.2)
Individual social needs			
≥1 of 4 Key social risks	4936 (36.5)	2631 (33.8)	2305 (40.1)
Financial stress (trouble paying for any basics)	3049 (22.5)	1613 (20.7)	1436 (25.0)
Food insecurity	2641 (19.5)	1384 (17.8)	1257 (21.9)
Housing barriers	2080 (15.4)	1123 (14.4)	957 (16.7)
Transportation barriers	1689 (12.5)	833 (10.7)	856 (14.9)
Health care use, mean (SD)			
Hospital admission	0.2 (0.6)	0.2 (0.6)	0.2 (0.7)
ED visit count	0.9 (2.2)	0.7 (2.0)	1.1 (2.3)
Primary care visit count	2.1 (3.0)	2.0 (2.9)	2.2 (3.1)
Specialist visit count	3.0 (4.4)	3.0 (4.4)	3.2 (4.4)
Mental health visit count	2.2 (9.4)	2.3 (10.1)	1.9 (8.6)
Social worker visit count	1.1 (3.9)	1.0 (3.7)	1.2 (4.3)

Abbreviations: ED, emergency department; NDI, Neighborhood Deprivation Index.

^a^ Race and ethnicity data were obtained from the Kaiser Permanente Northern California electronic health records.

^b^ Includes Native American (grouped due to small sample sizes), unknown, and other race or ethnicity.

**Figure 1.**
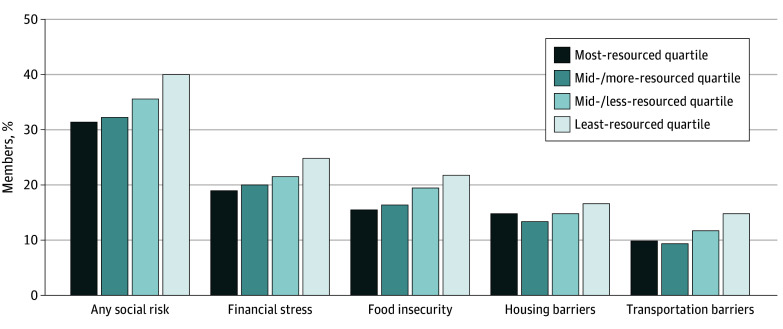
Self-Reported Social Risk by Neighborhood Deprivation Index Quartile

Overall, 36.5% of members reported one or more social risk. Self-report of one or more social risks ranged from 33.8% of members in the most-resourced quartile to 40.1% in the least-resourced quartile (*P* < .001). The most common social risk was financial stress (22.5%), followed by food insecurity (19.5%), housing barriers (15.4%), then transportation barriers (12.5%) (Figure 1). Differences in self-reported social risk between the more-resourced and least-resourced neighborhood quartiles encompassed a 4.5-percentage point spread for financial stress (20.7% to 25.0%), a 4.2-percentage point spread for transportation (10.7% to 14.9%), a 4.1-percentage point spread for food insecurity (17.8% to 21.9%), and a 2.3-percentage point spread for housing (14.4% to 16.7%). More on the prevalence of each self-reported social risk by neighborhood quartile is available in eTable 2 in Supplement 1.

**Figure 2.**
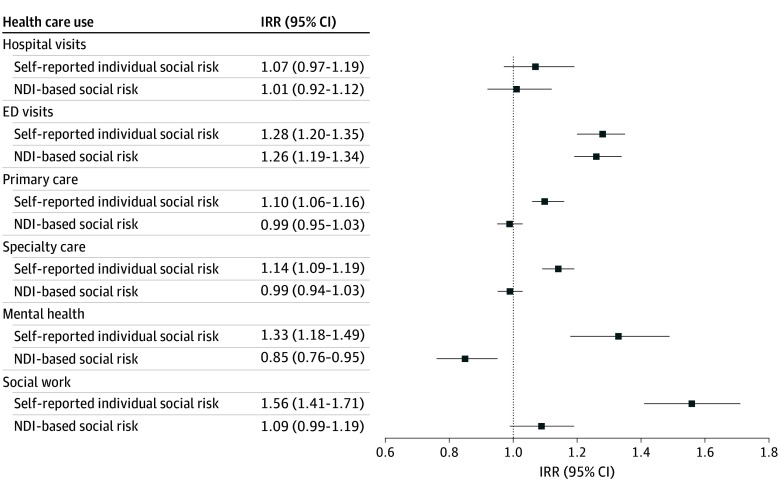
Health Care Use Association With Any Self-Reported Social Risk vs Neighborhood Deprivation Index–Based Risk NDI indicates Neighborhood Deprivation Index.

In the final multivariate models, which included both neighborhood and individual social risk measures and controlled for member demographic characteristics and health conditions, living in the least-resourced neighborhood quartile was not associated with differences in hospital visits (IRR, 1.01; 95% CI, 0.92-1.12), nor was having any individual social risk (IRR, 1.07; 95% CI, 0.97-1.19) (Table 3, model 3; Figure 2). Both neighborhood and individual indicators of social risk were associated with a greater risk of ED visits (neighborhood measure IRR, 1.26; 95% CI, 1.19-1.34; a marginal effect estimate of 0.23 visits; 95% CI, 0.17-0.29; and individual-measure IRR, 1.28; 95% CI, 1.20-1.35; a marginal effect estimate of 0.24 visits; 95% CI, 0.18-0.30).

**Table 3.** Multivariate Regression Results for Individual and Neighborhood Social Risk Associations With Health Care Use

Independent variables	IRR (95% CI)^a^			Model 3: Marginal effect estimates, difference in visits (95% CI)^a^
	Model 1: NDI only	Model 2: Any social risk only	Model 3: NDI and any social risk	
**Hospital visits**				
NDI most deprivation quartile (vs others)	1.01 (0.92 to 1.17)	NA	1.01 (0.92 to 1.12)	0.002 (−0.019 to 0.023)
Any social risk (vs none)	NA	1.07 (0.97 to 1.19)	1.07 (0.97 to 1.19)	0.015 (−0.006 to 0.037)
**ED visits**				
NDI	1.27 (1.20 to 1.34)	NA	1.26 (1.19 to 1.34)	0.233 (0.174 to 0.292)
Any social risk	NA	1.28 (1.20 to 1.36)	1.28 (1.20 to 1.35)	0.242 (0.182 to 0.303)
**Primary care**				
NDI	0.99 (0.95 to 1.03)	NA	0.99 (0.95 to 1.03)	−0.022 (−0.108 to 0.065)
Any social risk	NA	1.11 (1.06 to 1.16)	1.10 (1.06 to 1.16)	0.219 (0.131 to 0.308)
**Specialty care**				
NDI	0.99 (0.94 to 1.04)	NA	0.99 (0.94 to 1.03)	−0.040 (−0.185 to 0.107)
Any social risk	NA	1.14 (1.09 to 1.19)	1.14 (1.09 to 1.19)	0.415 (0.264 to 0.567)
**Mental health**				
NDI	0.86 (0.77 to 0.96)	NA	0.85 (0.76 to 0.95)	−0.688 (−1.191 to −0.184)
Any social risk	NA	1.32 (1.17 to 1.48)	1.33 (1.18 to 1.49)	1.208 (0.665 to 1.751)
**Social work**				
NDI	1.10 (0.99 to 1.21)	NA	1.09 (0.99 to 1.19)	0.131 (−0.125 to 0.274)
Any social risk	NA	1.56 (1.42 to 1.71)	1.56 (1.41 to 1.71)	0.666 (0.504 to 0.828)

Abbreviations: IRR, incident rate ratio; NA, not applicable; NDI, Neighborhood Deprivation Index.

^a^ All models were controlled for demographic characteristics (sex, age, race and ethnicity, interpreter need) and health conditions (tobacco use; anemia; asthma; chronic kidney disease; diabetes; hyperlipidemia; hypertension; rheumatoid arthritis and osteoarthritis; fibromyalgia, chronic pain, and fatigue; migraines; obesity; peripheral vascular disease; mental health disorders [depression, anxiety, personality disorder, posttraumatic stress disorder, bipolar disorder, and schizophrenia or schizoaffective conditions]; and substance use disorders [alcohol, opioid, and other drug use disorders]).

In contrast, comparisons between neighborhood or individual social risk diverged for other types of health care use. While neighborhood measurement was not associated with primary care use, individual measurement was associated with greater primary care use (IRR, 1.10; 95% CI, 1.06-1.16; a marginal effect estimate of 0.22 visits; 95% CI, 0.13-0.31). The same pattern held for specialty care and social work. For mental health, living in the least-resourced neighborhood quartile was associated with fewer mental health visits (IRR, 0.85; 95% CI, 0.76-0.95; a marginal effect estimate of −0.69 visits; 95% CI, −1.19 to −0.18), although individual social risk, as for other outpatient care, was associated with greater visits (IRR, 1.33; 95% CI, 1.18-1.49; marginal effect estimate, 1.21 additional visits; 95% CI, 0.67-1.75). Results in interim models (1 and 2) were consistent.

Analyses for each social risk instead of the composite any social risk also yielded directionally equivalent results. Incident rate ratios were significantly higher between transportation risk and ED visits (IRR, 1.41; 95% CI, 1.31-1.53), mental health visits (IRR, 1.76; 95% CI, 1.50-2.07), and social work visits (IRR, 1.90; 95% CI, 1.68-2.17) than the association between the any social risk composite and these types of health care visits (ED visits IRR, 1.28; 95% CI 1.20-1.35; mental health visits IRR, 1.33; 95% CI 1.18-1.49; social work IRR, 1.56; 95% CI 1.41-1.71). (eTable 3 in Supplement 1).

## Discussion

In our analyses comparing neighborhood- vs individual-level indicators of social risk, we found concordant associations with hospital visits and ED use, with both neighborhood and individual social risks associated with greater ED use. However, in contrast to our hypothesis, patterns of association diverged for outpatient primary care, specialty care, mental health, and social work visits. That is, while individual-level social risk was associated with greater outpatient health care use, neighborhood-level social risk was not associated with most outpatient care and was negatively associated with mental health visits.

Some findings are consistent with earlier work that has observed greater ED use in association with individual- and neighborhood-based social risk.^46,47,48^ We add to prior work by examining associations with ED use in the same population and find a similar outcome for both individual and neighborhood measures. Meanwhile, individual social risk has been associated with hospital admissions in some studies but not others.^47,48^ Our study’s finding of no association at both the neighborhood and individual levels suggests that other factors, including immediate clinical conditions, are more salient for hospitalization for respondents in our sample. As social risk–oriented health care interventions and new programs grow, often with a focus on reducing preventable health care use, the concordance of neighborhood and individual social risk indicators in relation to hospital and ED visits suggests that, in certain contexts, individual- and neighborhood-based social risk measurement may operate interchangeably, for example, in models to identify a population of focus.

Yet, it will be important for program designers to recognize that substituting neighborhood-based indicators of risk for individual risk would not correctly estimate the need for primary care, other specialty care including mental health, and social work services. As many states move to incorporate neighborhood-based measures of social risk into payment adjustment,^49^ incentives should ensure robust outpatient care access. Furthermore, linkages to ongoing preventive health services in less-resourced neighborhoods should be prioritized to ensure strong care quality. Past work finding less care management among patients in disadvantaged neighborhoods, including lower blood pressure control, cholesterol control, and diabetes-specific performance, offers an important caution.^50,51^ Our findings also suggest that improved access to mental health services in lower-resourced neighborhoods may be especially needed, as corroborated by recent evidence of lower mental health treatment and substance use disorder treatment in lower-resourced neighborhoods.^52^

Our findings also suggest that individual social risk screenings, more than neighborhood-based risk, may provide a stronger signal of potential health-compromising vulnerabilities and health care–seeking patterns across conditions, including mental health and social work service, than neighborhood indices. Even still, individual screening in our sample and others may underestimate social risk prevalence. While the prevalence of any social risk in our sample (36.5%) falls within the range of estimates from an extensive review of social risk screening literature (range, 8%-66%; median, 34%),^53^ some evidence indicates that people experiencing social risks report greater discomfort with screening than those without social risks.^54^ To enhance screening acceptability and accuracy, screenings should leverage trusting relationships with health care team members and include clear communication about the connection between social risks, member health, and the health services that members receive.^55,56^

### Limitations

Our results must be interpreted in the context of our study design. The sample includes members of a large integrated health system, and therefore results may look different in other health systems where services are less coordinated. In addition, the sample includes only Medicaid respondents (ie, individuals who meet a low-income threshold). Results may differ among people with a wider income range. Furthermore, approximately 50% of the enrollees responded to the Integrated Outcomes Questionnaire, and a direct comparison of respondents with all members offered the questionnaire is not possible, limiting our ability to assess selection bias. Possibly, nonrespondents feel less connected to health care services, and they may experience even greater social risks than responding members. Some evidence on social risk questionnaires indicates that nonrespondents have longer durations since their last primary care visit and lower perceptions of primary care.^54^ Consequently, our findings may reflect higher outpatient use than for the underlying Medicaid population.

In addition, opportunities for future research remain. Examining specific neighborhood features instead of a neighborhood-level index could yield new insights. Also, a prospective longitudinal design could contribute to better understanding of potential causality, as the current cross-sectional design identifies associations between social risks and health care use but does not permit inference about their temporal sequence.

## Conclusions

In this cross-sectional study comparing individual and neighborhood social risk measures, associations were concordant for hospital and ED use, but not outpatient visits. Given the great burden of collecting and managing individual-level social risk data, our analysis suggests that decision-makers primarily concerned with acute care use, such as ED visits, may use neighborhood-level indicators of social risk as a reasonable proxy. Nevertheless, screening for individual social risk during Medicaid enrollment yields valuable information that is distinct from neighborhood-level measurement. Relying on neighborhood-level measurement would likely underrepresent key outpatient health service needs, especially for mental health support.

## Notes

Supplement 1. **eTable 1.** Demographics by Presence of Self-Reported Social Risk **eTable 2.** Self-Reported Social Risks by Neighborhood Deprivation Index Quartile **eTable 3.** Associations Between Healthcare Use and Neighborhood-Based or Individual-Level Social Risks (Featuring Specific Risks) **eTable 4.** Characteristics of Integrated Outcomes Questionnaire Respondents Versus Kaiser Permanente Northern California Medicaid Members With at Least One Healthcare Visit

Supplement 2.
Data Sharing Statement

## References

[1] World Health Organization. Commission on Social Determinants of Health. Closing the gap in a generation: health equity through action on the social determinants of health: final report of the Commission on Social Determinants Of Health. August 27, 2008. Accessed February 21, 2025. https://www.who.int/publications/i/item/WHO-IER-CSDH-08.1

[2] Adler NE, Boyce WT, Chesney MA, Folkman S, Syme SL. Socioeconomic inequalities in health: no easy solution. JAMA. 1993;269(24):3140-3145. 8505817

[3] Marmot MG, Shipley MJ. Do socioeconomic differences in mortality persist after retirement? 25 year follow up of civil servants from the first Whitehall study. BMJ. 1996;313(7066):1177-1180. 8916748 10.1136/bmj.313.7066.1177 PMC2352486

[4] Braveman P, Egerter S, Williams DR. The social determinants of health: coming of age. Annu Rev Public Health. 2011;32:381-398. 21091195 10.1146/annurev-publhealth-031210-101218

[5] DeVoe JE, Bazemore AW, Cottrell EK, . Perspectives in primary care: a conceptual framework and path for integrating social determinants of health into primary care practice. Ann Fam Med. 2016;14(2):104-108. 26951584 10.1370/afm.1903 PMC4781512

[6] Adler NE, Cutler DM, Fielding JE, . Addressing social determinants of health and health disparities: a vital direction for health and health care. NAM Perspect. Published online September 19, 2016. https://nam.edu/wp-content/uploads/2016/09/Addressing-Social-Determinants-of-Health-and-Health-Disparities.pdf

[7] Joint Commission. Assess health-related social needs. Accessed May 3, 2024. https://www.jointcommission.org/our-priorities/health-care-equity/accreditation-resource-center/assess-health-related-social-needs/#t=_StrategiesTab&sort=%40created%20descending

[8] Centers for Medicare and Medicaid Services Measures Inventory Tool. Accessed July 3, 2024. https://cmit.cms.gov/cmit/#/FamilyView?familyId=1664

[9] Alderwick H, Gottlieb LM. Meanings and misunderstandings: a social determinants of health lexicon for health care systems. Milbank Q. 2019;97(2):407-419. 31069864 10.1111/1468-0009.12390 PMC6554506

[10] Brown MC, Lewis CC, Wellman RD, . 2022 Kaiser Permanente National Social Health Survey. July 2023. Accessed February 21, 2025. https://www.kpwashingtonresearch.org/application/files/9916/9833/8109/KP-National-Social-Health-Survey_2022_Quant-Results_Final-Report.pdf

[11] Zulman DM, Maciejewski ML, Grubber JM, . Patient-reported social and behavioral determinants of health and estimated risk of hospitalization in high-risk Veterans Affairs patients. JAMA Netw Open. 2020;3(10):e2021457. 33079198 10.1001/jamanetworkopen.2020.21457 PMC7576406

[12] Chi W, Andreyeva E, Zhang Y, Kaushal R, Haynes K. Neighborhood-level social determinants of health improve prediction of preventable hospitalization and emergency department visits beyond claims history. Popul Health Manag. 2021;24(6):701-709. 34010058 10.1089/pop.2021.0047

[13] Canterberry M, Figueroa JF, Long CL, . Association between self-reported health-related social needs and acute care utilization among older adults enrolled in Medicare Advantage. JAMA Health Forum. 2022;3(7):e221874. 35977222 10.1001/jamahealthforum.2022.1874 PMC9270697

[14] Ryan JL, Franklin SM, Canterberry M, . Association of health-related social needs with quality and utilization outcomes in a Medicare Advantage population with diabetes. JAMA Netw Open. 2023;6(4):e239316. 37083665 10.1001/jamanetworkopen.2023.9316 PMC10122170

[15] Fraze TK, Brewster AL, Lewis VA, Beidler LB, Murray GF, Colla CH. Prevalence of screening for food insecurity, housing instability, utility needs, transportation needs, and interpersonal violence by US physician practices and hospitals. JAMA Netw Open. 2019;2(9):e1911514. doi:10.1001/jamanetworkopen.2019.11514 31532515 PMC6752088

[16] Brewster AL, Rodriguez HP, Murray GF, Lewis VA, Schifferdecker KE, Fisher ES. Trends in screening for social risk in US physician practices. JAMA Netw Open. 2025;8(1):e2453117. doi:10.1001/jamanetworkopen.2024.53117 39752156 PMC11699528

[17] Ackerman SL, Wing H, Aceves B, Pisciotta M, Hessler D, Gottlieb LM. “We were trying to do quality versus quantity”: Challenges and opportunities at the intersection of standardized and personalized social care in community health centers. SSM Qual Res Health. 2023;3:100267. doi:10.1016/j.ssmqr.2023.100267

[18] Rogers AJ, Hamity C, Sharp AL, Jackson AH, Schickedanz AB. Patients’ attitudes and perceptions regarding social needs screening and navigation: multi-site survey in a large integrated health system. J Gen Intern Med. 2020;35(5):1389-1395. doi:10.1007/s11606-019-05588-1 31898132 PMC7210366

[19] De Marchis EH, Hessler D, Fichtenberg C, . Part I: a quantitative study of social risk screening acceptability in patients and caregivers. Am J Prev Med. 2019;57(6)(suppl 1):S25-S37. doi:10.1016/j.amepre.2019.07.010 31753277 PMC7336892

[20] Cullen D, Wilson-Hall L, McPeak K, Fein J. Pediatric social risk screening: leveraging research to ensure equity. Acad Pediatr. 2022;22(2):190-192. doi:10.1016/j.acap.2021.09.013 34571253 PMC8479442

[21] Butler ED, Morgan AU, Kangovi S. Screening for unmet social needs: patient engagement or alienation? Catal Non-Issue Content. 2020;1(4). doi:10.1056/CAT.19.1037

[22] Garg A, Boynton-Jarrett R, Dworkin PH. Avoiding the unintended consequences of screening for social determinants of health. JAMA. 2016;316(8):813-814. doi:10.1001/jama.2016.9282 27367226

[23] Messer LC, Laraia BA, Kaufman JS, . The development of a standardized neighborhood deprivation index. J Urban Health. 2006;83(6):1041-1062. doi:10.1007/s11524-006-9094-x 17031568 PMC3261293

[24] Zhang Y, Ancker JS, Hall J, Khullar D, Wu Y, Kaushal R. Association between residential neighborhood social conditions and health care utilization and costs. Med Care. 2020;58(7):586-593. doi:10.1097/MLR.0000000000001337 32520834

[25] Cubbin C, Egerter S, Braveman P, Pedregon V. Where we live matters for our health: neighborhoods and health. October 2008. Accessed October 27, 2023. https://folio.iupui.edu/handle/10244/638

[26] Silva M, Loureiro A, Cardoso G. Social determinants of mental health: a review of the evidence. Eur J Psychiatry. 2016;30(4):259-292.

[27] Pickett KE, Pearl M. Multilevel analyses of neighbourhood socioeconomic context and health outcomes: a critical review. J Epidemiol Community Health. 2001;55(2):111-122. doi:10.1136/jech.55.2.111 11154250 PMC1731829

[28] Brown EM, Franklin SM, Ryan JL, . Assessing area-level deprivation as a proxy for individual-level social risks. Am J Prev Med. 2023;65(6):1163-1171. doi:10.1016/j.amepre.2023.06.006 37302512

[29] Miller-Rosales C, McCloskey J, Uratsu CS, Ralston JD, Bayliss EA, Grant RW. Associations between different self-reported social risks and neighborhood-level resources in Medicaid patients. Med Care. 2022;60(8):563-569. doi:10.1097/MLR.0000000000001735 35640038 PMC9262842

[30] Bensken WP, McGrath BM, Gold R, Cottrell EK. Area-level social determinants of health and individual-level social risks: assessing predictive ability and biases in social risk screening. J Clin Transl Sci. 2023;7(1):e257. doi:10.1017/cts.2023.680 38229891 PMC10790234

[31] Cottrell EK, Hendricks M, Dambrun K, . Comparison of community-level and patient-level social risk data in a network of community health centers. JAMA Netw Open. 2020;3(10):e2016852. doi:10.1001/jamanetworkopen.2020.16852 33119102 PMC7596576

[32] Powers BW, Figueroa JF, Canterberry M, . Association between community-level social risk and spending among Medicare beneficiaries: implications for social risk adjustment and health equity. JAMA Health Forum. 2023;4(3):e230266. doi:10.1001/jamahealthforum.2023.0266 37000433 PMC10066453

[33] Beckett MK, Martino SC, Agniel D, . Distinguishing neighborhood and individual social risk factors in health care. Health Serv Res. 2022;57(3):458-471. doi:10.1111/1475-6773.13884 34596232 PMC9108057

[34] Morenz AM, Liao JM, Au DH, Hayes SA. Area-level socioeconomic disadvantage and health care spending: a systematic review. JAMA Netw Open. 2024;7(2):e2356121. doi:10.1001/jamanetworkopen.2023.56121 38358740 PMC10870184

[35] Medicaid Waiver tracker. Approved and pending Section 1115 waivers by state. Accessed August 22, 2024. https://www.kff.org/medicaid/issue-brief/medicaid-waiver-tracker-approved-and-pending-section-1115-waivers-by-state/

[36] Davis AC, Voelkel JL, Remmers CL, Adams JL, McGlynn EA. Comparing Kaiser Permanente members to the general population: implications for generalizability of research. Perm J. 2023;27(2):87-98. doi:10.7812/TPP/22.172 37170584 PMC10266863

[37] von Elm E, Altman DG, Egger M, Pocock SJ, Gøtzsche PC, Vandenbroucke JP; STROBE Initiative. The Strengthening the Reporting of Observational Studies in Epidemiology (STROBE) statement: guidelines for reporting observational studies. Lancet. 2007;370(9596):1453-1457. doi:10.1016/S0140-6736(07)61602-X 18064739

[38] Ross TR, Ng D, Brown JS, . The HMO Research Network Virtual Data Warehouse: a public data model to support collaboration. EGEMS (Wash DC). 2014;2(1):1049. doi:10.13063/2327-9214.1049 25848584 PMC4371424

[39] Sundar KR. Universal screening for social needs in a primary care clinic: a quality improvement approach using the your current life situation survey. Perm J. 2018;22(4S):18-089. doi:10.7812/TPP/18-089 30296397 PMC6175598

[40] SIREN. Your current life situation survey. Accessed August 1, 2024. https://sirenetwork.ucsf.edu/tools-resources/resources/your-current-life-situation-survey

[41] Lewis CC, Wellman R, Jones SMW, . Comparing the performance of two social risk screening tools in a vulnerable subpopulation. J Family Med Prim Care. 2020;9(9):5026-5034. doi:10.4103/jfmpc.jfmpc_650_20 33209839 PMC7652127

[42] Diez Roux AV. Neighborhoods and health: what do we know? what should we do? Am J Public Health. 2016;106(3):430-431. doi:10.2105/AJPH.2016.303064 26885960 PMC4815954

[43] Akinyemi O, Weldeslase T, Odusanya E, . The relationship between neighborhood economic deprivation and asthma-associated emergency department visits in Maryland. Front Allergy. 2024;5:1381184. doi:10.3389/falgy.2024.1381184 38903705 PMC11188351

[44] Roy AM, George A, Attwood K, . Effect of neighborhood deprivation index on breast cancer survival in the United States. Breast Cancer Res Treat. 2023;202(1):139-153. doi:10.1007/s10549-023-07053-4 37542631 PMC10504126

[45] Vos AA, Posthumus AG, Bonsel GJ, Steegers EAP, Denktaş S. Deprived neighborhoods and adverse perinatal outcome: a systematic review and meta-analysis. Acta Obstet Gynecol Scand. 2014;93(8):727-740. doi:10.1111/aogs.12430 24834960

[46] Carlson LC, Kim J, Samuels-Kalow ME, . Comparing neighborhood-based indices of socioeconomic risk factors and potentially preventable emergency department utilization. Am J Emerg Med. 2021;44:213-219. doi:10.1016/j.ajem.2020.03.035 32291162

[47] Mosen DM, Banegas MP, Keast EM, Dickerson JF. Examining the association of social needs with future health care utilization in an older adult population: which needs are most important? Popul Health Manag. 2023;26(6):413-419. doi:10.1089/pop.2023.0171 37943589 PMC10698796

[48] Clennin M, Schootman M, Reifler L, Tavel H, Cromwell L. Association of social risk factors with emergency department and inpatient hospitalization encounters before and during the COVID-19 pandemic. 2022. Accessed July 17, 2024. https://kpwashingtonresearch.org/application/files/4016/7639/1403/SONNET_Report_Social-Risk-Health-Care-Utilization_FINAL.pdf

[49] Robert L. Phillips J, Ostrovsky A, Gilfillan R, Price D, Bazemore AW. Accounting for social risks in Medicare and Medicaid payments. Health Affairs. March 27, 2023. Accessed February 21, 2025. https://www.healthaffairs.org/content/forefront/accounting-social-risks-medicare-and-medicaid-payments

[50] Durfey SNM, Kind AJH, Buckingham WR, DuGoff EH, Trivedi AN. Neighborhood disadvantage and chronic disease management. Health Serv Res. 2019;54(suppl 1)(suppl 1):206-216. doi:10.1111/1475-6773.13092 30468015 PMC6341202

[51] Kurani SS, Lampman MA, Funni SA, . Association between area-level socioeconomic deprivation and diabetes care quality in US primary care practices. JAMA Netw Open. 2021;4(12):e2138438. doi:10.1001/jamanetworkopen.2021.38438 34964856 PMC8717098

[52] Gibbons RD, Olfson M, Saulsberry L, . Social vulnerability and prevalence and treatment for mental health and substance use disorders. JAMA Psychiatry. 2024;81(10):976-984. July 24, 2024. doi:10.1001/jamapsychiatry.2024.1870 39046728 PMC11446668

[53] De Marchis EH, Brown E, Aceves B, . SCREEN report: state of the science of social screening in healthcare settings. SIREN: Social Interventions Research and Evaluation Netowrk. 2022. Accessed February 3, 2025. https://sirenetwork.ucsf.edu/sites/default/files/2022-06/final%20SCREEN%20State-of-Science-Report%5B55%5D.pdf

[54] Ray KN, Gitz KM, Hu A, Davis AA, Miller E. Nonresponse to health-related social needs screening questions. Pediatrics. 2020;146(3):e20200174. doi:10.1542/peds.2020-0174 32753371

[55] Papajorgji-Taylor D, Hsu C, Mosen D. Assessing members’ experiences and preferences on social needs and how to receive support: qualitative results from the 2022 Kaiser Permanente National Social Health Survey Final Report. June 2023. Accessed February 21, 2025. https://kpwashingtonresearch.org/application/files/7516/9833/8383/KP-National-Social-Health-Survey_2022_Qual-Results_Final-Report.pdf

[56] Brown EM, Loomba V, De Marchis E, Aceves B, Molina M, Gottlieb LM. Patient and patient caregiver perspectives on social screening: a review of the literature. J Am Board Fam Med. 2023;36(1):66-78. doi:10.3122/jabfm.2022.220211R1 36759136

