# Supplementary material for: Social Risk at Individual vs Neighborhood Levels and Health Care Use in Medicaid Enrollees
Source: JAMA Netw Open. 2025 Apr 15;8(4):e255047. doi: 10.1001/jamanetworkopen.2025.5047 (PMC12000970; doi:10.1001/jamanetworkopen.2025.5047)
Supplement: Supplement 1. — eTable 1. Demographics by Presence of Self-Reported Social Risk eTable 2. Self-Reported Social Risks by Neighborhood Deprivation Index Quartile eTable 3. Associations Between Healthcare Use and Neighborhood-Based or Individual-Level Social Risks (Featuring Specific Risks) eTable 4. Characteristics of Integrated Outcomes Questionnaire Respondents Versus Kaiser Permanente Northern California Medicaid Members With at Least One Healthcare Visit [file jamanetwopen-e255047-s001.pdf]

Supplemental Online Content

Knox MJ, Tucher EL, Miller-Rosales C, McCloskey J, Grant RW, Iturralde E. Social risk at individual vs neighborhood levels and health care use in Medicaid enrollees. *JAMA Netw Open.* 2025;8(4):e255047. doi:10.1001/jamanetworkopen.2025.5047

eTable 1. Demographics by Presence of Self-Reported Social Risk

eTable 2. Self-Reported Social Risks by Neighborhood Deprivation Index Quartile

eTable 3. Associations Between Healthcare Use and Neighborhood-Based or Individual-Level Social Risks (Featuring Specific Risks)

eTable 4. Characteristics of Integrated Outcomes Questionnaire Respondents Versus Kaiser Permanente Northern California Medicaid Members With at Least One Healthcare Visit

This supplemental material has been provided by the authors to give readers additional information about their work.

© 2025 Knox MJ et al. J Netw Open.

eTable 1: Demographics by presence of self-reported social risk

Characteristic	**Total** (Members, No. [%])	**Self-reported social risks = none** (Members, No. [%])	**Self-reported social risks = 1 or more** (Members, No. [%])
	N=13,593	N=11,506	N=2,087
Demographics			
Sex			
Female	8,648 (63.8)	5,293 (61.5)	3,355 (67.8)
Male	4,906 (36.2)	3,313 (38.5)	1,593 (32.2)
Age, by category			
18-24	1,872 (13.8)	1,338 (15.5)	534 (10.8)
25-44	5,303 (39.1)	3,535 (41.1)	1,768 (35.7)
45-64	4,262 (31.4)	2,375 (27.6)	1,887 (38.1)
65+	2,117 (15.6)	1,358 (15.8)	759 (15.3)
Race/Ethnicity			
Asian	4,611 (34.0)	2,996 (34.8)	1,615 (32.6)
Black/African American	2,854 (21.1)	2,034 (23.6)	820 (16.6)
Hispanic	1,988 (14.7)	939 (10.9)	1,049 (21.2)
White	3,045 (22.5)	1,936 (22.5)	1,109 (22.4)
Other[a]	1,056 (7.8)	701 (8.1)	355 (7.2)
Needs Interpreter	1,151 (8.5)	784 (9.1)	367 (7.4)
Tobacco use: current use	1,487 (11.0)	713 (8.3)	774 (15.6)
Diagnoses			
Anemia	1,398 (10.3)	750 (8.7)	648 (13.1)
Asthma	2,111 (15.6)	1,103 (12.8)	1,008 (20.4)
Chronic Kidney Disease	1,369 (10.1)	693 (8.1)	676 (13.7)
Diabetes	2,294 (16.9)	1,254 (14.6)	1,040 (21.0)
Hyperlipidemia	2,954 (21.8)	1,663 (19.3)	1,291 (26.1)
Hypertension	3,793 (28.0)	2,084 (24.2)	1,709 (34.5)
Acquired Hypothyroidism	986 (7.3)	575 (6.7)	411 (8.3)
Rheumatoid Arthritis/Osteoarthritis	1,818 (13.4)	933 (10.8)	885 (17.9)
Fibromyalgia, Chronic Pain, and Fatigue	2,368 (17.5)	1,074 (12.5)	1,294 (26.2)
Migraine and Chronic Headache	1,208 (8.9)	596 (6.9)	612 (12.4)
Obesity	2,681 (19.8)	1,387 (16.1)	1,294 (26.2)
Peripheral Vascular Disease	1,569 (11.6)	846 (9.8)	723 (14.6)
Depression	2,693 (19.9)	1,237 (14.4)	1,456 (29.4)
Anxiety	2,694 (19.9)	1,329 (15.4)	1,365 (27.6)
Personality disorder	363 (2.7)	159 (1.8)	204 (4.1)
Bipolar disorder	731 (5.4)	335 (3.9)	396 (8.0)

© 2025 Knox MJ et al. J Netw Open.

**Characteristic**	**Total** (Members, No. [%])	**Self-reported social risks = none** (Members, No. [%])	**Self-reported social risks = 1 or more** (Members, No. [%])
Post-traumatic stress disorder	382 (2.8)	127 (1.5)	255 (5.2)
Schizophrenia or schizo-affective conditions	432 (3.2)	219 (2.5)	213 (4.3)
Alcohol use disorder	293 (2.2)	127 (1.5)	166 (3.4)
Drug or opioid use disorder	1,069 (7.9)	463 (5.4)	606 (12.2)
Individual Social Needs			
One or More out of 4 key social risks	4,948 (36.5)	0 (0.0)	4,948 (100.0)
Financial Stress (Trouble Paying for Any Basics)	3,059 (22.6)	0 (0.0)	3,059 (61.8)
Food Insecurity	2,648 (19.5)	0 (0.0)	2,648 (53.5)
Housing Barriers	2,085 (15.4)	0 (0.0)	2,085 (42.1)
Transportation barriers	1,692 (12.5)	0 (0.0)	1,692 (34.2)
Neighborhood Deprivation Index Quartile			
Most Resourced	1,521 (11.2)	1,040 (12.1)	481 (9.7)
Mid-High Resourced	2,485 (18.4)	1,680 (19.6)	805 (16.3)
Mid-Low Resourced	3,775 (27.9)	2,430 (28.3)	1,345 (27.2)
Least Resourced	5,746 (42.5)	3,441 (40.1)	2,305 (46.7)
Healthcare Use	*Mean (SD)*	*Mean (SD)*	*Mean (SD)*
Hospital Admission	0.9 (2.2)	0.7 (1.9)	1.3 (2.4)
ED Visit Count	0.2 (0.6)	0.2 (0.6)	0.3 (0.8)
Primary Care Visit Count	2.1 (3.0)	1.7 (2.5)	2.6 (3.6)
Specialist Visit Count	3.0 (4.4)	2.6 (3.9)	3.8 (5.1)
Mental Health Visit Count	2.2 (9.4)	1.7 (8.9)	3.0 (10.3)
Social Worker Visit Count	1.1 (3.9)	0.7 (3.0)	1.7 (5.1)

© 2025 Knox MJ et al. J Netw Open.

eTable 2: Self-Reported Social Risks by Neighborhood Deprivation Index Quartile

Social Risk	Total	Most resourced	Mid-high resourced	Mid-low resourced	Least resourced
	N=13,527	N=1,521	N=2,485	N=3,775	N=5,746
One or more of 4 key social risks	4,936 (36.5%)	481 (31.6%)	805 (32.4%)	1345 (35.6%)	2,305 (40.1%)
Financial Stress	3,049 (22.5%)	291 (19.1%)	499 (20.1%)	823 (21.8%)	1,436 (25.0%)
Food Insecurity	2,641 (19.5%)	237 (15.6%)	412 (16.6%)	735 (19.5%)	1,257 (21.9%)
Housing Barriers	2,080 (15.4%)	228 (15.0%)	332 (13.4%)	563 (14.9%)	957 (16.7%)
Transportation barriers	1,689 (12.5%)	152 (10.0%)	236 (9.5%)	445 (11.8%)	856 (14.9%)

© 2025 Knox MJ et al. J Netw Open.

eTable 3 - Associations Between Healthcare Use and Neighborhood-Based or Individual-Level Social Risks (Featuring Specific Risks)

Independent Variable	Any of 4 Social Risks	Financial Risk	Food Insecurity	Housing	Transportation
	IRR (95% CI)	*IRR (95% CI)*	*IRR (95% CI)*	*IRR (95% CI)*	*IRR (95% CI)*
**Hospital Visits**					
NDI most deprivation quartile (vs. others)	1.01 (0.92 – 1.12)	1.01 (0.92-1.12)	1.01 (0.92 – 1.12)	1.01 (0.92 – 1.12)	1.01 (0.92 – 1.12)
Social risk present (vs. not)	1.07 (0.97 – 1.19)	1.01 (0.91-1.13)	0.97 (0.86 – 1.09)	1.01 (0.89 – 1.14)	1.08 (0.95 – 1.23)
**ED Visits**					
NDI	1.26 (1.19 – 1.34)	1.27 (1.19-1.34)	1.26 (1.19 – 1.34)	1.27 (1.20 – 1.35)	1.26 (1.19 – 1.34)
Social risk present	1.28 (1.20 - 1.35)	1.23 (1.15-1.31)	1.21 (1.13 - 1.29)	1.24 (1.15 - 1.34)	1.41 (1.31 - 1.53)
**Primary Care**					
NDI	0.99 (0.95 – 1.03)	0.99 (0.95 – 1.03)	0.99 (0.95 – 1.03)	0.99 (0.95 – 1.03)	0.99 (0.95 – 1.03)
Social risk present	1.10 (1.06 – 1.16)	1.08 (1.02 – 1.13)	1.10 (1.04 – 1.15)	1.07 (1.01 – 1.12)	1.07 (1.01 – 1.13)
**Specialty Care**					
NDI	0.99 (0.94 – 1.03)	0.99 (0.94 – 1.03)	0.99 (0.94 – 1.03)	0.99 (0.95 – 1.04)	0.99 (0.94 – 1.03)
Social risk present	1.14 (1.09 – 1.19)	1.13 (1.07 - 1.19)	1.05 (0.99 – 1.12)	1.11 (1.05 – 1.18)	1.10 (1.03 – 1.18)
**Mental Health**					
NDI	0.85 (0.76 – 0.95)	0.86 (0.77 – 0.97)	0.85 (0.76 – 0.96)	0.85 (0.76 – 0.95)	0.85 (0.76 – 0.95)
Social risk present	1.33 (1.18 – 1.49)	1.16 (1.02 – 1.33)	1.20 (1.04 – 1.38)	1.66 (1.44 – 1.92)	1.76 (1.50 – 2.07)
**Social Work**					
NDI	1.09 (0.99 – 1.19)	1.09 (0.99 – 1.20)	1.10 (1.00 – 1.21)	1.11 (1.01 – 1.21)	1.09 (0.99 – 1.20)
Social risk present	1.56 (1.41 – 1.71)	1.51 (1.36 – 1.67)	1.41 (1.26 – 1.57)	1.52 (1.35 – 1.71)	1.90 (1.68 – 2.17)

All models include both neighborhood-based and individual social risk and control for demographics (sex, age, race, interpreter need) and health conditions (tobacco use, anemia, asthma, chronic kidney disease, diabetes, hyperlipidemia, hypertension, rheumatoid arthritis/osteoarthritis, fibromyalgia /chronic pain/ fatigue, migraines, obesity, mental health disorders [depression, anxiety, personality disorder, post-traumatic stress disorder, bipolar disorder, and schizophrenia or schizo-affective conditions], and substance use disorders [alcohol, opioid, and other drug use disorders]).

© 2025 Knox MJ et al. J Netw Open.

eTable 4: Characteristics of Integrated Outcomes Questionnaire Respondents Versus Kaiser Permanente Northern California Medicaid Members with at Least One Healthcare Visit

Note – The Integrated Outcomes Questionnaire was administered for operational and not research purposes, thus response rates were not systematically tracked. Basic demographic traits of Medicaid members with at least on outpatient visit during a similar timeframe as the study period are provided below as a proxy to compare survey respondents to the broader membership offered the questionnaire.

Demographic Characteristics	***Study Sample***	***KPNC Medicaid (2018-2020, 1+ outpatient visit)***
	N=13,527	***N = 125,252***
Women	*8,631 (63.8%)*	*64.5%*
Age, by category		
18-24	*1,870 (13.8%)*	*23.9%*
25-44	*5,289 (39.1%)*	*43.1%*
45-64	*4,256 (31.5%)*	*25.3%*
65+	*2,112 (15.6%)*	*7.7%*
Race/Ethnicity		
Asian	*2,846 (21.0%)*	*17.0%*
Black/African American	*1,986 (14.7%)*	*15.4%*
Hispanic	*3,040 (22.5%)*	*28.9%*
White	*4,602 (34.0%)*	*33.6%*
Other	*1,053 (7.8%)*	*5.1%*

© 2025 Knox MJ et al. J Netw Open.
