# Supplementary material for: Social Risk at Individual vs Neighborhood Levels and Health Care Use in Medicaid Enrollees
Source: JAMA Netw Open. 2025 Apr 15;8(4):e255047. doi: 10.1001/jamanetworkopen.2025.5047 (PMC12000970; doi:10.1001/jamanetworkopen.2025.5047)
Supplement: Supplement 2. — Data Sharing Statement [file jamanetwopen-e255047-s002.pdf]

Data Sharing Statement

Knox. Social Risk at Individual vs Neighborhood Levels and Health Care Use in Medicaid Enrollees. *JAMA Netw Open*. Published April 15, 2025.
doi:10.1001/jamanetworkopen.2025.5047

Data
Data available: No

Additional Information
Explanation for why data not available: Please contact the corresponding author for data inquiries.
